# Upper nasal hemifield location and nonspatial auditory tones accelerate visual detection during dichoptic viewing

**DOI:** 10.1371/journal.pone.0199962

**Published:** 2018-07-23

**Authors:** Terhi Mustonen, Mikko Nuutinen, Lari Vainio, Jukka Häkkinen

**Affiliations:** Department of Psychology and Logopedics, University of Helsinki, Helsinki, Finland; Moorfields Eye Hospital NHS Foundation Trust, UNITED KINGDOM

## Abstract

Visual performance is asymmetric across the visual field, but locational biases that occur during dichoptic viewing are not well understood. In this study, we characterized horizontal, vertical and naso-temporal biases in visual target detection during dichoptic stimulation and explored whether the detection was facilitated by non-spatial auditory tones associated with the target’s location.

The detection time for single monocular targets that were suppressed from view with a 10 Hz dynamic noise mask presented to the other eye was measured at the 4° intercardinal location of each eye with the breaking Continuous Flash Suppression (b-CFS) technique. Each target was either combined with a sound (i.e., high or low pitch tone) that was congruent or incongruent with its vertical location (i.e., upper or lower visual field) or presented without a sound. The results indicated faster detection of targets in the upper rather than lower visual field and faster detection of targets in the nasal than temporal hemifield of each eye. Sounds generally accelerated target detection, but the tone pitch-elevation congruency did not further enhance performance. These findings suggest that visual detection during dichoptic viewing differs from standard viewing conditions with respect to location-related perceptual biases and crossmodal modulation of visual perception. These differences should be carefully considered in experimental designs employing dichoptic stimulation techniques and in display applications that utilize dichoptic viewing.

## Introduction

Visual perception is asymmetric across the visual field. Visual performance is known to generally decrease with increasing distance from the fovea due to losses in the contrast sensitivity and spatial resolution of the eye [1], but performance consistently varies across the visual field, even at isoeccentric locations. Indeed, a number of studies demonstrate performance differences for equidistant stimuli presented in the left and right visual fields, upper and lower visual fields, and temporal and nasal hemifields of the eyes [2–12]. However, because these performance asymmetries have been defined under standard viewing conditions, in which stimuli are viewed either binocularly or monocularly, the results do not necessarily comply with dichoptic viewing, during which both eyes are simultaneously presented with different stimuli. The present study aims to clarify this largely unexplored topic by investigating spatial biases in target detection during dichoptic viewing and the influence of crossmodal cueing on detection performance with a dichoptic stimulation technique known as Continuous Flash Suppression (CFS; [13]).

When an observer’s right and left eye are presented with dissimilar images at corresponding retinal locations, the images cannot be fused and the conscious percept varies between the two alternatives. This phenomenon, known as *binocular rivalry*, is characterized as a random alternation of dominance and suppression phases between the two eyes [14]. At a given moment during dichoptic viewing, the observer only perceives the stimulus presented to the dominant eye, whereas the stimulus presented to the suppressed eye remains outside conscious perception. Although the mechanisms that control the alteration of the suppression and dominance phases are not fully understood [14,15], research has shown that the ability of a stimulus to break suppression, in other words, to become detected, is strongly defined by low-level visual features, such as contrast, orientation, and spatial frequency [16,17]. However, another fundamental feature that is likely to modify the stimulus strength under dichoptic stimulation is the location of the target in the visual field.

Research on location-based biases in visual performance suggest that horizontal, vertical, and naso-temporal asymmetries originate from different stages of the visual processing stream. Most horizontal asymmetries (left vs. right visual field) reflect the lateral specialization of the two hemispheres. Because the left hemisphere is more sensitive to local features and high spatial frequencies and the right hemisphere is sensitive to global features and low spatial frequencies, high-frequency stimuli are more likely to bias perception rightwards and low-frequency stimuli are more likely to bias perception leftwards [3,18]. A horizontal bias may also indicate an uneven distribution of spatial attention, as performance in many attention-demanding visual tasks is biased toward the left side of space [19]. This leftward bias may result from the higher involvement of the right hemisphere in tasks that require visuospatial attention [20] or the left-to-right scanning strategy that resembles reading text in Western languages [21].

Vertical asymmetries (upper vs. lower visual field) greatly depend on the requirements of the visual task. Stimulus discrimination is more efficient in the lower than upper visual field [5,8,11,12], which is in line with anatomical asymmetries in the retina [22,23] and lateral geniculate nucleus (LGN) [24]. A lower visual field bias has also been found for tasks that require focused sustained attention, including conjunction search and multiple object tracking [25], as well as attentional tasks that require object individuation [26]. In contrast to these downward biases, an upward bias is common for tasks that require attention guidance, such as a visual search [7,27], or semantic processing, including categorical judgments [6,28]. It thus appears that subcortical and early cortical visual processes tend to bias perception downwards, whereas attention may bias performance either downwards or upwards depending on the task demands.

**Fig 1:**
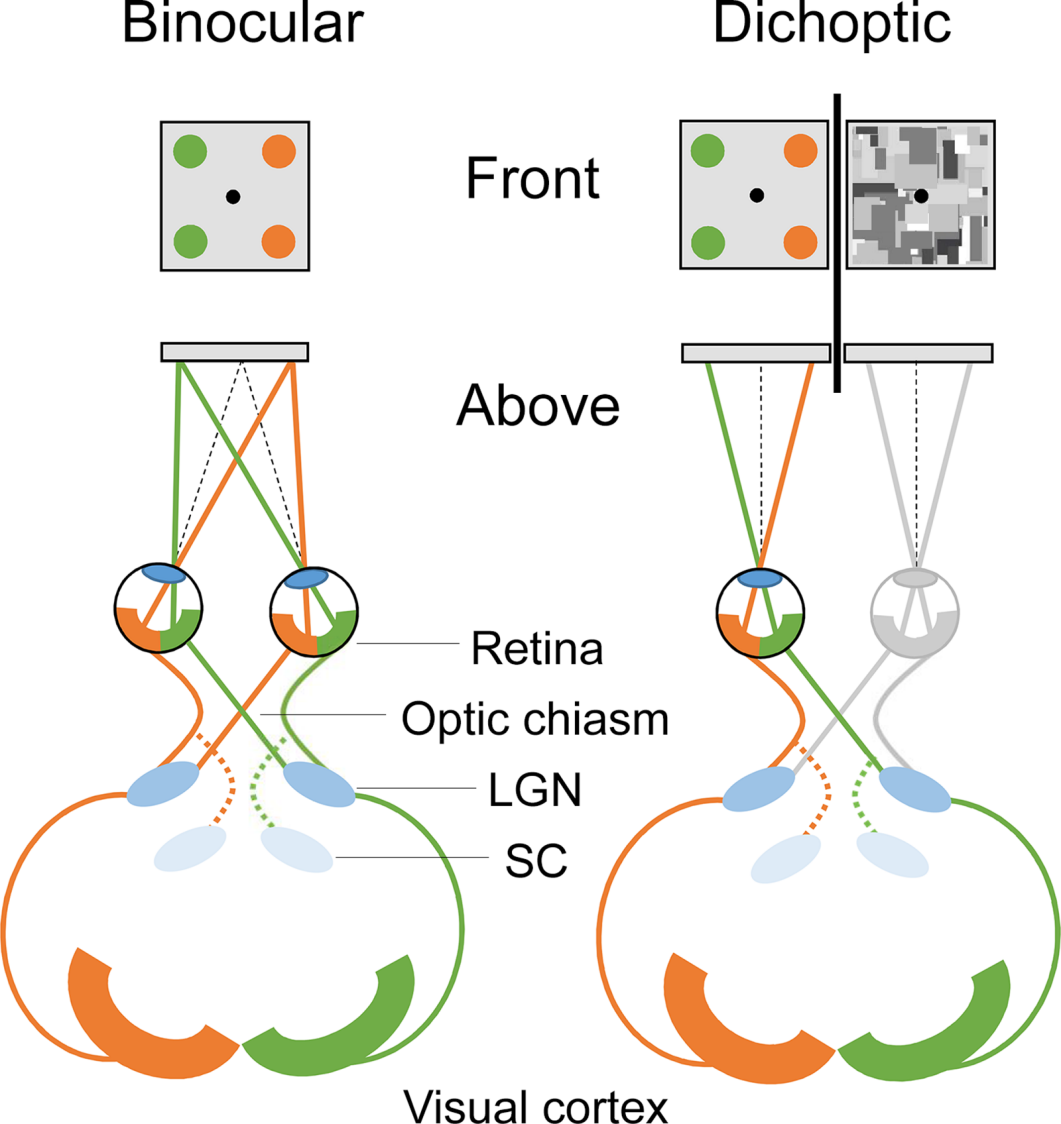
Lateralized visual processing in standard and dichoptic viewing. In standard binocular viewing (left side), targets in the left visual field (green) are projected onto the right hemiretina (nasal or temporal) of both eyes and processed in the right hemisphere. Targets in the right hemifield (orange) are similarly processed in the left hemisphere. Each hemisphere, therefore, receives information from both eyes via ipsilateral and contralateral connections. Under dichoptic viewing (right side), targets in the temporal hemifield of an eye (green) are projected onto the nasal hemiretina of that eye and processed in the contralateral hemisphere and targets in the nasal hemifield (orange) are projected onto the temporal hemiretina and processed in the ipsilateral hemisphere. Lateralized targets in a dichoptic viewing task are thus processed in one hemisphere only, which may change the typical course of visual processing.

However, the most intriguing asymmetry in the context of the present study is the bias between the retinal hemifields (nasal vs. temporal) as the direction of the bias under standard viewing conditions seems to contradict the findings of dichoptic viewing. Under standard viewing conditions, visual processing is more efficient in the temporal hemifield than nasal hemifield of the eye, as demonstrated by sensitivity thresholds [10,12], reaction times [9], and electrophysiological recordings [12]. This temporal hemifield bias is associated with anatomical asymmetries in the retina [22,23] and superior colliculus (SC) of the midbrain [29]. By contrast, two binocular rivalry studies have previously demonstrated longer dominance durations for stimuli presented to the nasal hemifield of an eye (i.e., temporal hemiretina; Fig 1) compared to stimuli presented to the temporal hemifield (i.e., nasal hemiretina) [30,31] under dichoptic stimulation. Because the nasal hemifield bias was not predicted by asymmetries in early visual structures, Chen and He [30] and Kaushall [31] suggest that the bias likely reflects a cortical origin and thus differs from the temporo-nasal bias obtained under standard viewing conditions. The present study aims to investigate whether the nasal hemifield bias associated with dichoptic stimulation is specific for extending dominance durations under rivalry alternation or whether it also facilitates detection of suppressed visual targets under strong interocular suppression. Similarly, detection biases for suppressed visual targets along the vertical and horizontal axes are characterized.

In addition to clarifying location-based asymmetries in target detection during dichoptic viewing, we aim to take a further step by investigating whether this process is facilitated by an auditory cue associated with the target’s location. A strong body of evidence indicates that visual objects combined with an auditory signal are detected, localized and identified more rapidly and accurately than visual objects alone [32]. Although most of these findings concern the spatial and temporal compatibility of visual and auditory stimuli, recent findings suggest that crossmodal facilitation may also operate on non-spatial associative *crossmodal correspondences* [33–35]. Importantly, in the present study, one such correspondence occurs between an auditory tone pitch and visual elevation. Responses to visual targets that appear higher in the visual field are detected faster when the targets are combined with a concurrent high pitch tone than with a low pitch tone, and targets lower in the visual field are detected faster when combined with a low pitch tone than with a high pitch tone [36–38]. Under standard viewing conditions, pitch-elevation compatibility has been suggested to support the processing of visual stimuli at both perceptual [37] and response selection levels [33,36], even when the observer is unaware of the connection between the two signals [37]. Whether the compatibility of a non-spatial auditory sound to a visual target—a connection typically obtained with two supraliminal stimuli—can similarly accelerate the detection of a suppressed visual target during dichoptic viewing has not yet been investigated.

However, other audiovisual effects suggest that crossmodal modulations can also occur under dichoptic stimulation. From the two competing stimuli presented to an observer’s eyes during binocular rivalry, the stimulus compatible with a concurrent sound with respect to frequency [39], directional motion [40], or semantic content [41] dominates perception most of the time. Furthermore, previous b-CFS studies have demonstrated that a visual stimulus that matches information simultaneously presented via another modality can break the suppression faster than a mismatching stimulus [42]. The connection between visual and auditory stimuli can be physical [43], spatial [44], or semantic [45] in nature. On the other hand, not all findings similarly support the integration of visual and auditory stimuli. Moors and colleagues [46] investigated whether the detection of suppressed visual looming targets could be influenced by auditory tone pips that were congruent with the looming cycle. By measuring contrast thresholds for target detection, they found no evidence that auditory tones lowered detection thresholds compared to no-sound trials [46]. Previous studies, thus, suggest that some but not all forms of crossmodal modulations emerge under interocular suppression. By demonstrating that the pitch of an auditory tone facilitates the detection of suppressed visual targets whose location (in the upper or lower visual field) is associated with the pitch of the given tone, the present study not only provides further support for the automaticity of crossmodal correspondences [34,37] but also introduce a new means to modulate location-specific visual processing during dichoptic viewing.

### Present study

The goals of the present study were twofold. First, we aimed to characterize visual field asymmetries in the detection of suppressed visual targets during dichoptic stimulation. Second, we explored whether this detection process could be facilitated by an auditory tone associated with the target’s vertical location in the upper or lower visual field.

For these purposes, we employed the CFS technique, in which a stimulus (i.e., the target) presented to one eye is deliberately rendered invisible by a strong suppressor (i.e., dynamic noise) simultaneously presented to the other eye [13]. In CFS, a conflict between the two retinal images creates a strong rivalry condition in which the observer only perceives the suppressor and not the target, even though both stimuli are physically present. The CFS thus represents an evolved version of the binocular rivalry paradigm in which both eyes are simultaneously presented with different images and each eye’s dominance periods are reported during natural perceptual alternation [14]. Because the strong suppressor utilized in CFS elevates target detection thresholds over 10-fold compared to binocular rivalry [13,17], even a highly salient target remains invisible for an extended period of time. This process makes the CFS particularly suitable for studying factors that drive the alteration between the two competing views.

Visual targets, suppressed from view by the CFS noise mask presented to the other eye, were displayed at four intercardinal locations of each eye. Therefore, we were able to compare the performance for targets presented in the two horizontal (right vs. left), vertical (upper vs. lower), and hemiretinal (nasal vs. temporal) locations of the visual field. To clarify whether the target detection could be modulated by associated sounds, each target in two of three experimental blocks was combined with a simple tone (i.e., high or low pitch) that was either compatible or incompatible with the target’s vertical location (i.e., upper or lower visual field). The targets in the third block were presented without sounds to provide a baseline for visual performance without crossmodal effects. Performance was defined as the time required for target detection with a procedure known as “breaking CFS” (b-CFS) [47]. This procedure provides a means to explore visual and audiovisual effects on target detection within a single design that closely resembles faster classification tasks under standard viewing conditions [32,34].

Based on previous dichoptic stimulation studies [30,31], we expected to find a nasal hemifield bias in the speed of target detection during the b-CFS task. No a priori assumptions were made concerning performance biases along the horizontal and vertical dimensions of space, but these biases were expected to provide valuable information about the demands of the dichoptic task and the processes involved in rivalry alteration. Regarding the crossmodal modulation of target detection during the b-CFS task, we expected the presence of sounds and tone pitch-target elevation congruency to increase speed of performance. Briefly, our results demonstrated the shortest b-CFS times for targets presented in the upper nasal hemifield of each eye and indicated that the presence of a sound but not pitch-location compatibility increased the speed of the detection performance.

## Method

### Subjects

The subjects were 30 students from the University of Helsinki (7 males; mean age = 24.7 ± 3.1 years) who were all right-handed (L.Q. > 62 in Edinburgh Handedness Inventory) [48]. All subjects reported normal hearing and normal or corrected-to-normal vision and wore normal corrective lenses during the experiment. Subjects were screened for stereopsis (TNO test for stereoscopic vision), heterophoria (Maddox Wing Test, Hamblin Instruments Ltd), and near distance visual acuity at three contrast levels (SLOAN near vision charts at 100%, 10%, and 2.5% contrast; Precision Vision®). Eighteen subjects were right-eye dominant, as determined by a hole-in-the-card test [49]. Eye dominance was controlled for in the statistical analysis due to its known effects on performance during dichoptic viewing [50]. Subjects gave written informed consent prior to the experiment and received two movie tickets as remuneration. This study was conducted with ethical approval of the University of Helsinki Ethical Review Board in Humanities and Social and Behavioral Sciences and adhered to the tenets of the Declaration of Helsinki.

### Apparatus and stimuli

Stimulus generation and presentation were controlled using MATLAB with the Psychophysics toolbox 3 extension [51]. An open-source collection of MATLAB functions that we created for this purpose are described in a previous paper [52]. Experiments were run on a PC with a Windows 7 operating system. Visual stimuli were displayed on a 21-in. Sony Trinitron CRT monitor (2048 x 1536; 75 Hz). Visual output was linearized with gamma correction (of 0.48) that was derived from gray level measurements captured by a Minolta LS-110 luminance meter. The monitor was viewed perpendicularly through a mirror stereoscope so that the left half of the screen was only seen by the left eye and right half was only seen by the right eye. An 83-cm viewing distance was maintained with a chin rest. Experiments were performed in a darkened room in which the only light source was the display.

**Fig 2:**
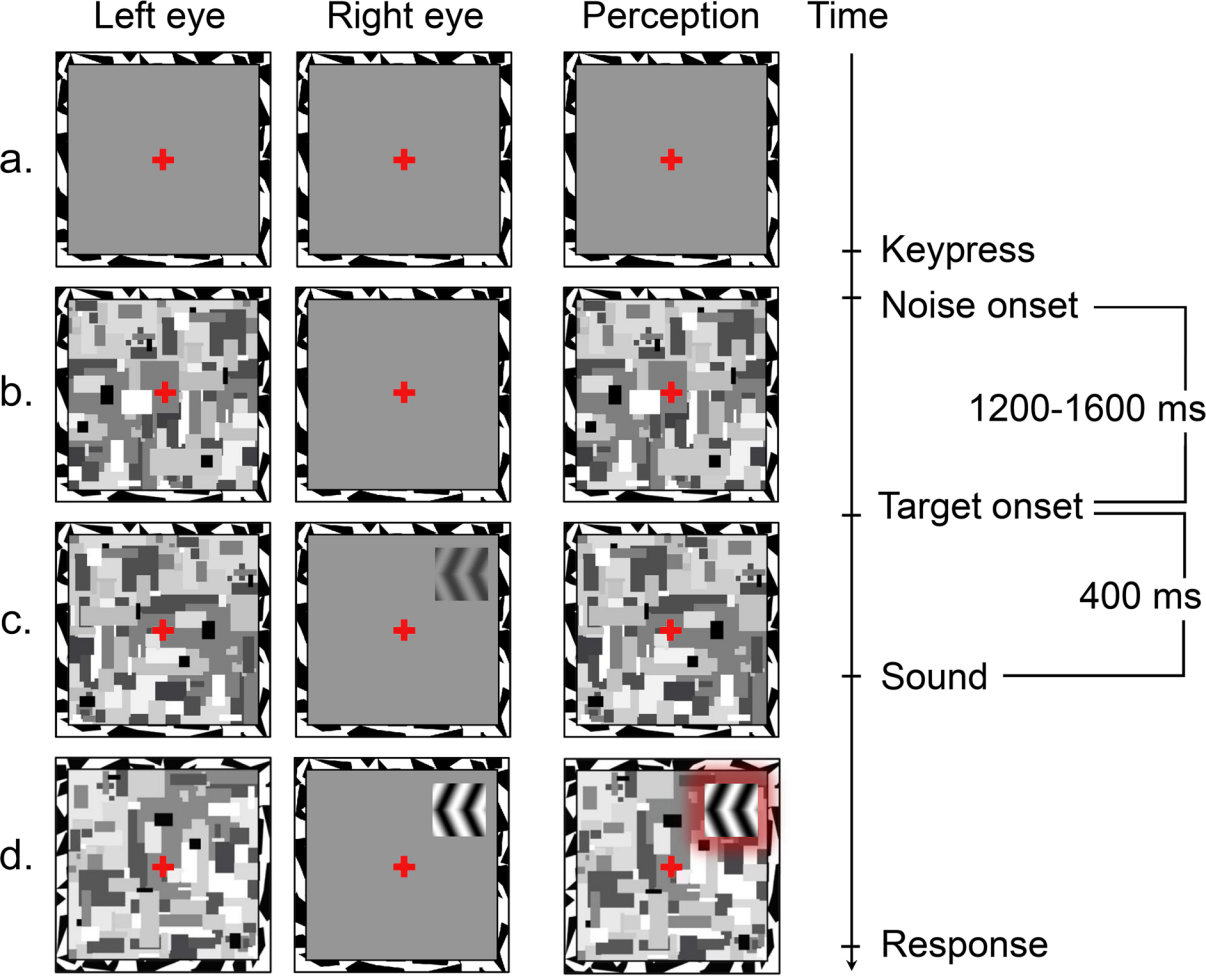
Schematic illustration of a trial. (a) The trial starts with a fixation cross presented to both eyes. The subject initiates the actual trial with a key press. (b) Dynamic Mondrian noise is presented to one of the eyes. (c-d) The target is presented to the other eye, and its contrast is gradually ramped up. The target remains until the subject’s response. When sound is included, a high or low pitch tone is provided for 400 ms after target onset.

Visual stimuli were displayed against two 9° gray squares (30 cd/m^2^) positioned 13.8° apart from each other to the left and right halves of the screen and surrounded by 0.5° wide black-and-white frames (Fig 2). The frames and red central fixation crosses (0.6°) were present at all times to promote stable binocular fusion. The farther background of the monitor was black (0.2 cd/m^2^). The suppressor stimulus consisted of a rapid series (10 Hz) of achromatic Mondrian-patterned images that filled one of the frames. Each image consisted of rectangles that varied in size, luminance and location. The mean luminance of the suppressor was (30 cd/m2), and its spatial characteristics followed the statistics of natural scenes (“Dead Leaves Model”) [53]. Target stimuli were achromatic leftwards and rightwards arrow shapes (1.5° x 1.5°; Fig 2C and 2D) that were generated from rectangular Gabor patches (1.5 cpd; 25° clockwise or counterclockwise orientation) by mirror-reversing the lower halves of the patches. The target characteristics were carefully controlled because the spatiotemporal properties of stimuli are known to affect the suppression strength in CFS [16]. A target appeared at one of four locations of the other frame (upper left, upper right, lower left, lower right) at a 4° eccentricity and 55° radial angle from the vertical meridian. These locations enabled unilateral presentation of the targets while avoiding eye movements that were needed for target detection at farther eccentricities [21,54].

The auditory stimuli were two sinusoidal tones: 1750 Hz for the high pitch and 250 Hz for the low pitch. The tones were presented using headphones at approximately 60 dB for 100 ms using the Psychtoolbox PsychPortAudio command library [51]. The high and low pitch tones were matched in subjective loudness according to ISO 226 equal-loudness contours [55].

### Procedure

Each trial began with a framed grey square presented to each eye and fused together (Fig 2). Subjects were instructed to fixate on the cross in the middle of the frame and indicate the stimulus detection by pressing the space key. The CFS mask then appeared in one of the frames. After a random delay of 1200–1600 ms, a target began to appear in the other frame. The eye of target presentation changed on each trial to avoid the effects of eye dominance and adaptation on detection performance [50,56]. None of the subjects was aware of this controlled sequence when asked after the experiment. The contrast of the target was gradually ramped up from 0 to 100% over 4000 ms to avoid an abrupt transition. The target remained until the response was given or timeout at 6000 ms. In sound-associated blocks, a high or low pitch tone was presented binaurally after 400 ms of target onset (for a similar procedure, see [44]). At this point, the target reached 10% contrast. Subjects’ ability to detect targets at this contrast level was validated with a pilot study. Subjects’ task was to respond to the target occurrence based on its horizontal location (i.e., left vs. right to the fixation cross). Upon detecting a target, subjects were instructed to press one of two colored keys on the keyboard: “e” with the left hand for targets on the left and “o” with the right hand for targets on the right. This indirect task in which the dimensions of response selection and response keys (i.e., left vs. right) were orthogonal to the dimension on which the expected crossmodal correspondence occurred (i.e., up/high vs. down/low) was adopted to avoid response selection bias in detection performance (see [37]). If the pitch-elevation congruency effect was to be found, it could not be accounted for by convergence at the decision or response level. Performance was measured as the reaction time (RT) using the b-CFS procedure [47].

The experiment consisted of three blocks of 160 trials each. Visual targets differed only with respect to location and orientation, which were counterbalanced within the blocks. In two of the blocks, visual targets were combined with mixed high and low auditory cues. No cues were used in the third block. Half of the subjects first completed the cued blocks and half the no-sound block. The block order was taken into account in statistical analysis to control for learning effects and because the block order may give rise to performance differences between the visual field areas [28]. High and low pitch tones appeared equally often with each of these combinations. Approximately ten minutes of training was given before the actual experiment. Each actual block was preceded by 20 warm-up trials that were removed from the final data.

## Results

Only correct response trials (99.3%) were included in the RT analysis. Very long or short RTs (beyond 3SDs from the subject’s mean within sound or no-sound trials) were considered to be outliers and removed from the data. In total, 2.5% of the original data were excluded. Because the distribution of the RTs was skewed, the dataset was log-transformed before any statistical tests. For the same reason, the descriptive statistics below represent the geometric means of the RT distribution produced by backward transformations of the analyzed data.

Data were analyzed using a mixed-design ANOVA with *Location* (upper left, upper right, lower left, lower right), *Eye* (left, right) and *Sound* (high sound, low sound, no sound) as within-subject factors and *Dominance* (left eye, right eye) and *Order* (sound first, no-sound first) as between-subject factors (S1 Table). A Greenhouse-Geisser correction was applied whenever Mauchly’s test indicated that sphericity could not be assumed. An alpha level of .05 was used for all statistical tests.

**Fig 3:**
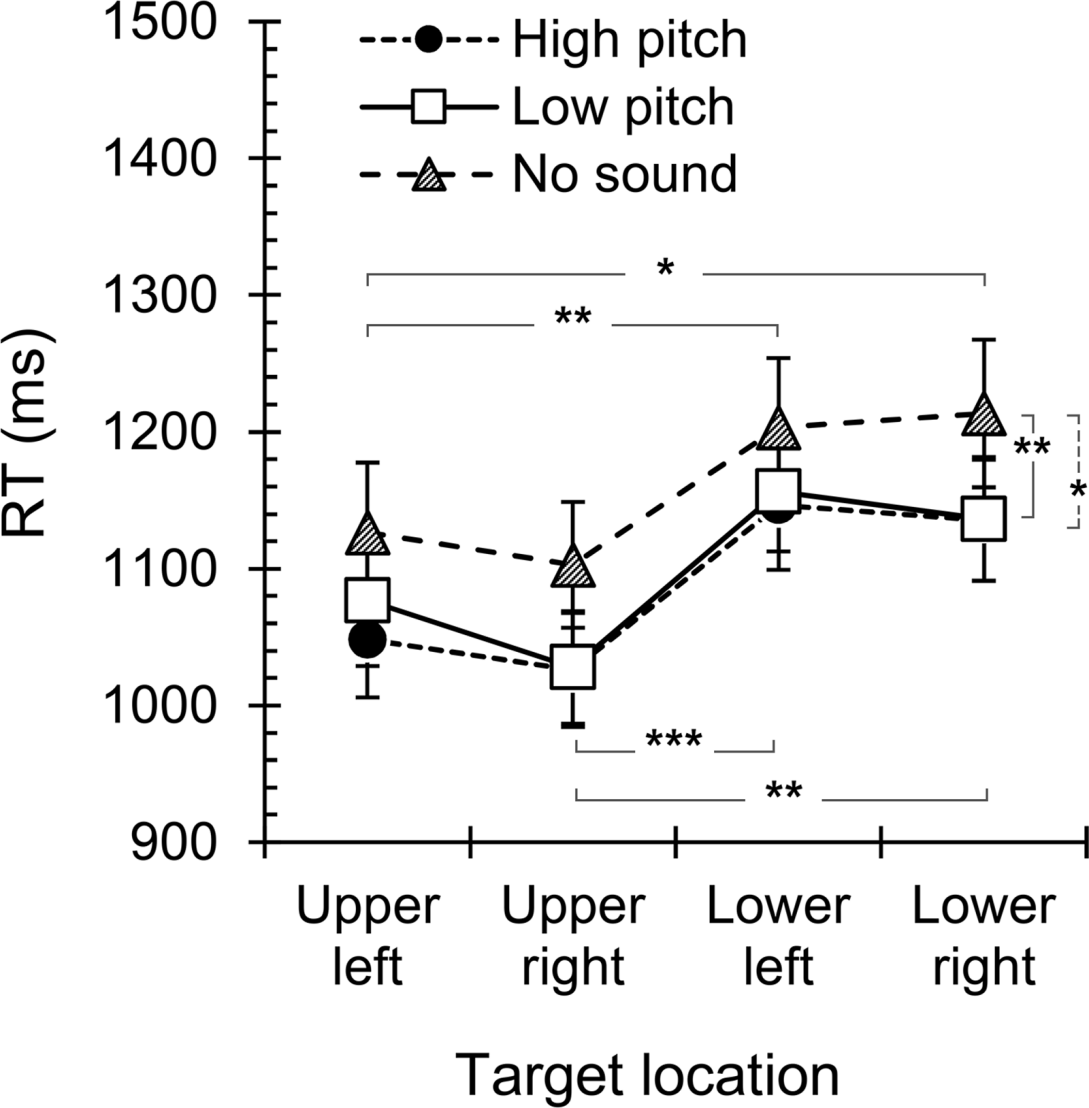
Effects of location and sound cue on the detection of suppressed visual targets. Mean b-CFS RTs for targets presented at four display quadrants when associated with high pitch tone, low pitch tone, or presented without a sound. Error bars represent the standard errors of the mean. * p < .05, ** p < .01, *** p < .001.

**Fig 4:**
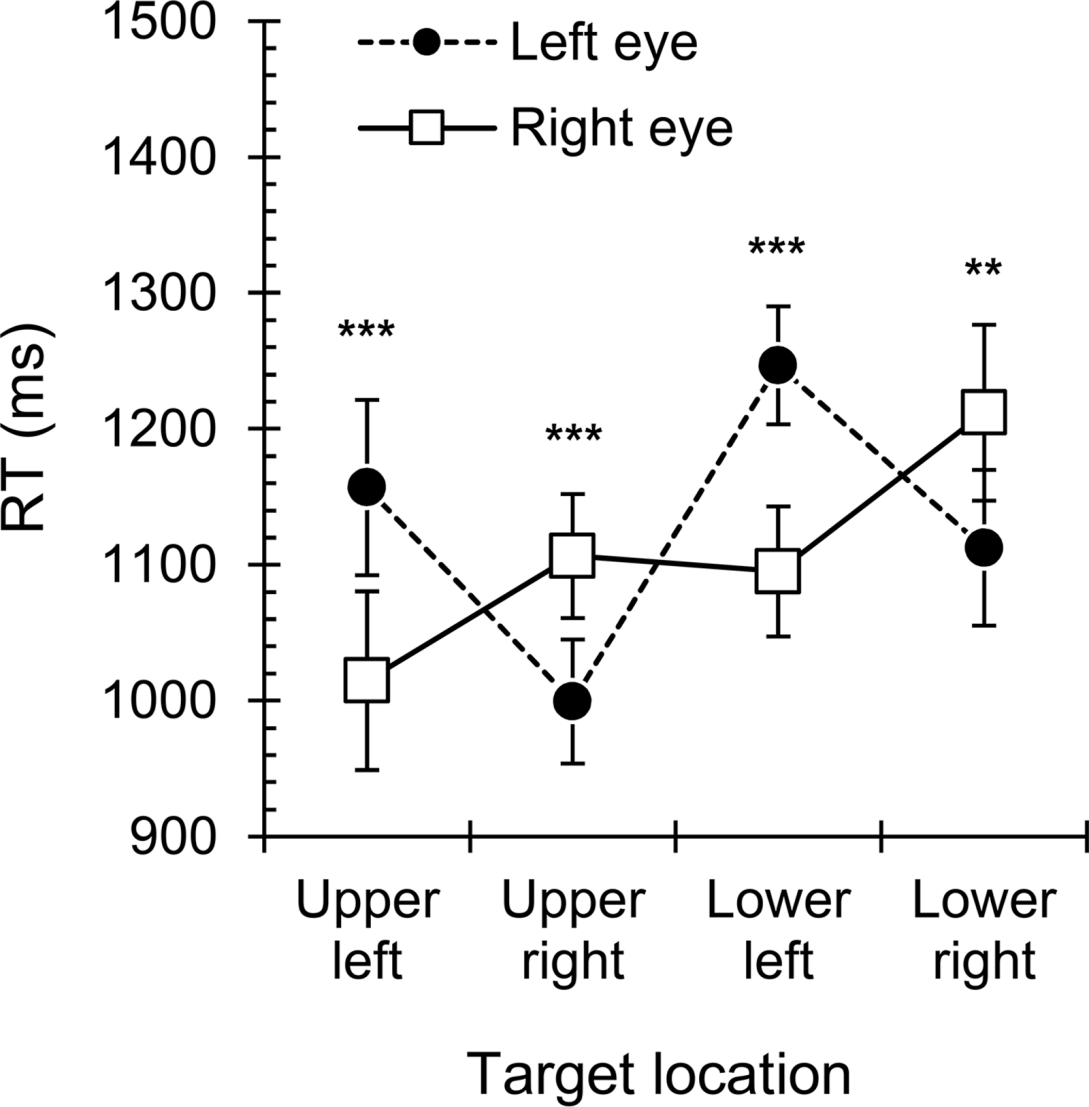
Location x eye interaction in the detection of suppressed visual targets. Mean b-CFS RTs for targets at four display locations in the left and right eye. Error bars represent the standard errors of the mean. ** p < .01, *** p < .001.

A significant main effect was found for *Location*, *F*(3, 78) = 10.9, *p* < .001, η_p_^2^ = .30. Bonferroni-adjusted post-hoc tests (six comparisons) revealed an upward vertical bias, as the targets presented in the upper quadrants of the visual field (left *M* = 1083 ms, 95% CI [980, 1197]; right *M* = 1052 ms, 95% CI [958, 1155]) were detected faster than the targets presented in the lower quadrants of the visual field (left *M* = 1168 ms, 95% CI [1064, 1283]; right *M* = 1161 ms, 95% CI [1056, 1277]; *p*_*adj*_ = .001- .041; Fig 3). Furthermore, the *Location x Eye* interaction, *F*(1.53, 39.8) = 21.4, *p* < .001, η_p_^2^ = .45, indicated that the time to detection also depended on the eye of presentation, as left-side targets resulted in shorter RTs when presented to the right eye and right-side targets had shorter RTs when presented to the left eye (Fig 4). Thus, targets presented to the nasal half of an eye’s visual field (i.e., temporal hemiretina) were detected faster than targets presented to the temporal hemifield (i.e., nasal hemiretina) of that eye.

**Fig 5:**
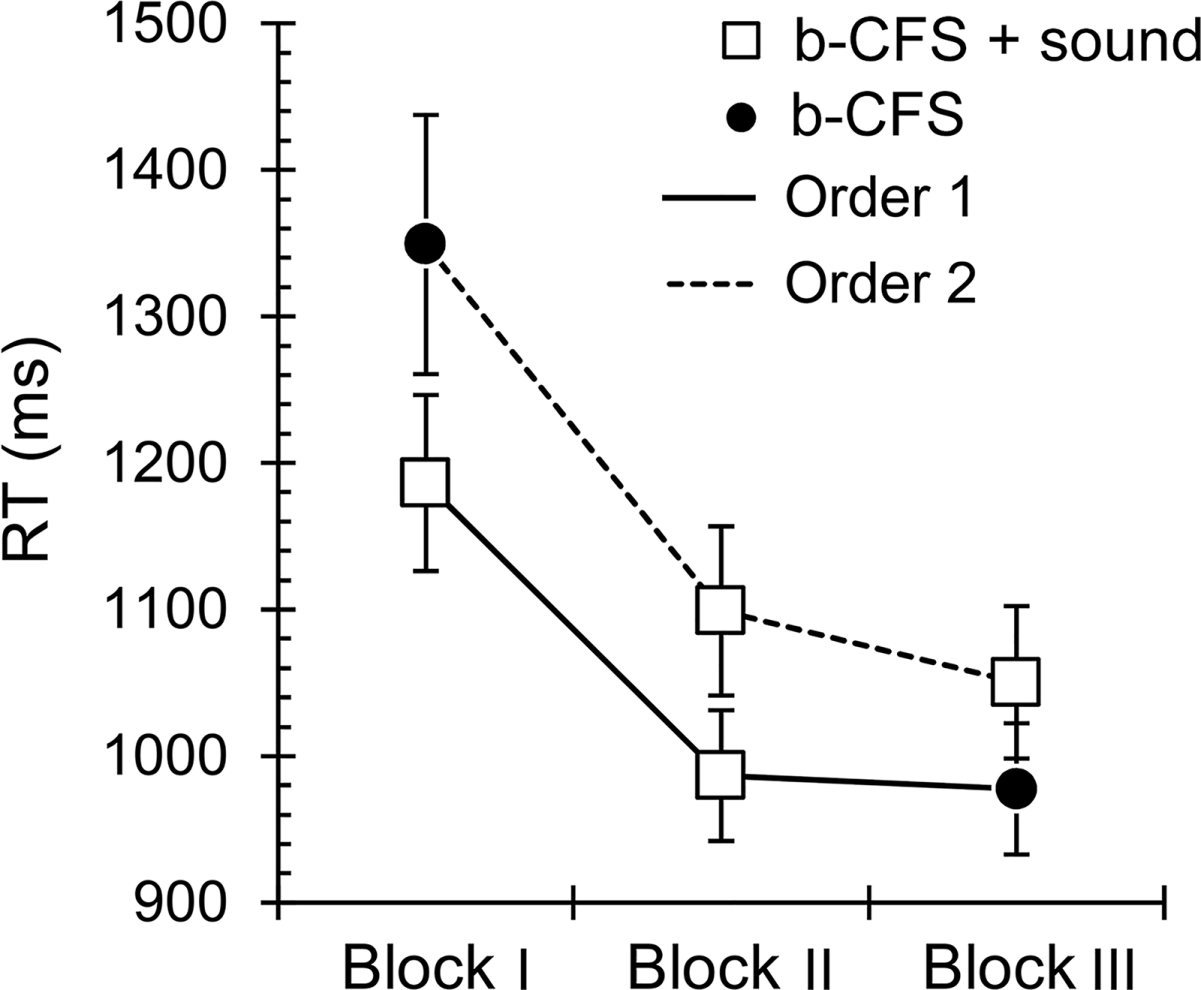
Sound-associated learning in the b-CFS task. Mean b-CFS RTs for three stimulus blocks (in order of presentation). Visual targets were combined with auditory tones in two of the blocks (open squares) and presented without sounds in one block (filled circles). The blocks were carried out in one of two orders so that the no-sound block was either the first block (solid line, n = 15) or last block (dashed line; n = 15) of the sequence. The presence of sounds was associated with an increased learning rate during the course of the experiment.

The main effect of *Sound* was also significant, *F*(1.16, 30.1) = 9.55, *p* = .003, η_p_^2^ = .27. Bonferroni-adjusted post-hoc tests (three comparisons) indicated that targets combined with a sound (high pitch tone *M* = 1088 ms, 95% CI [993, 1191]; low pitch tone *M* = 1098 ms, 95% CI [1002, 1204]) broke through suppression faster than no-sound targets (*M* = 1161 ms, 95% CI [1053, 1279]; *p*_*adj*_ = .005 - .027), but the RTs associated with high and low pitch tones did not differ (*p*_*adj*_ = .41; Fig 3). Notably, the *Sound x Location* interaction did not reach significance, *F*(4.05, 105) = .59, *p* = .67, η_p_^2^ = .022. This finding suggests that tone pitch-elevation compatibility did not facilitate the detection of suppressed visual targets during dichoptic stimulation. However, the *Sound x Order* interaction, *F*(1.16, 30.1) = 67.4, *p* < .001, η_p_^2^ = .73, indicated that the presence of auditory tones influenced learning during the experiment (Fig 5). The first b-CFS block always resulted in the longest RTs, independent of the presence of auditory tones, which suggests that the subjects became more proficient with the task over the course of the trials. Interestingly, RTs for sound-associated visual targets varied much less with trial iteration than RTs for non-sound targets, as the latter were slow in early trials but notably faster at the end of the experiment. This finding suggests that simple sounds associated with visual targets facilitated learning in the b-CFS task. No significant main effects were found for *Eye*, *F*(1, 26) = 3.18, *p* = .086, η_p_^2^ = .11; *Dominance*; *F*(1, 26) = .041, *p* = .84, η_p_^2^ = .002; or *Order*, *F*(1, 26) = 1.84, *p* = .19, η_p_^2^ = .07. All other interactions were also non-significant, *F* ≤ 3.18, *p* ≥ .09, η_p_^2^ ≤ .11.

## Discussion

To characterize visual field asymmetries for target detection during dichoptic viewing, we compared the b-CFS times for targets presented in the upper or lower, left or right, and nasal or temporal hemifield of each eye. Whether the detection process was modulated by simple auditory tones whose pitch (i.e., high or low) was or was not compatible with the vertical location of the targets (i.e., upper or lower visual field) was also explored. The results demonstrated that visual targets broke suppression faster when presented in the upper rather than lower visual field and faster in the nasal rather than temporal hemifield of the eye. The symmetric performance for targets in the left and right visual fields indicated that results were not confounded by factors such as strategic scanning [21] or spatial frequency content [3,18], as these factors were expected to bias performance along the horizontal dimension of space. Furthermore, simple auditory tones generally increased the speed of detection of suppressed visual targets, but tone pitch-target elevation congruency did not improve performance further. These findings suggest that visual processing during dichoptic viewing differs from standard viewing conditions with respect to perceptual biases and the crossmodal modulation of visual processing.

### Nasal hemifield bias and upward bias in b-CFS

The nasal hemifield bias in the b-CFS task contradicts the temporal hemifield bias that is characteristic of performance under standard viewing conditions [9–11]. However, the bias is in line with previous dichoptic stimulation studies that have demonstrated a nasal hemifield bias in dominance durations during binocular rivalry [30,31]. Chen and He [30] and Kaushall [31] suggested that this specific bias could reflect the altered dominance of crossed and uncrossed visual processing pathways during dichoptic stimulation. In normal binocular viewing, each hemifield is projected onto the nasal hemiretina of the ipsilateral eye and the temporal hemiretina of the contralateral eye (Fig 1). Both hemispheres thus receive information from the right and left visual fields. However, when the two eyes’ visual fields are separated, as in dichoptic viewing, each hemifield (of a given eye) is projected onto only one hemiretina and one hemisphere. In this case, the nasal hemifield of an eye (i.e., the temporal hemiretina) with an uncrossed connection to the ipsilateral hemisphere dominates perception over the temporal hemifield of that eye (i.e., the nasal hemiretina) with a crossed connection to the contralateral side. The nasal hemifield bias could thus result from the dominance of the uncrossed visual pathway over the crossed visual pathway under the specific case of dichoptic viewing [30,31]. Based on this interpretation, cortical connections that take place after the initial stimulus encoding play a greater role in defining visual performance biases in dichoptic viewing than the biased representation of the visual field in the retina and subcortical structures [22,23,29], which in turn, are supposed to be responsible for the temporal bias under standard viewing conditions. By demonstrating that the nasal hemifield bias is not limited to the binocular rivalry paradigm but that it also occurs for the strong interocular suppression induced by the CFS, the present study provides further support for this spatial asymmetry pattern, which is specific to dichoptic viewing.

Similarly, the upward bias in target detection during the b-CFS task contradicts the findings from the discrimination and localization tasks carried out in non-dichoptic viewing. Sensitivity for visual targets in such tasks is typically greater in the lower than upper visual field [5,8,11,12], which corresponds with the anatomical asymmetries in the retina and geniculate areas [22–24]. Instead of this early perceptual asymmetry, the upward bias is consistent with the vertical bias typically obtained in visual search tasks that require higher-order perceptual and attentional processing [6,7,27,28]. The b-CFS task employed in the present study might thus have operated as a dichoptic version of visual search, in which the dynamic noise mask presented to one distracted bottom-up driven processes related to the detection of the target presented to the other eye, thus requiring an increased top-down search for the target. In other words, given that people tend to orient their spatial attention towards the upper rather than lower visual field [6,7], it is possible that the current task boosted top-down attention toward the upper space before the target reached a threshold for conscious perception. This tendency might have led to faster responses for targets presented higher in the visual field in the present b-CFS task. Correspondingly, Kanai et al. [57] utilized a CFS task to demonstrate that the neural representation of suppressed visual stimuli can be modulated by top-down attention guidance. In support of our results, their findings suggested that attentional factors may contribute to the processing of visual stimuli, even in the absence of, or prior to, conscious perception [57].

However, one methodological constraint must be considered when generalizing the present results. From the RTs obtained in b-CFS experiments, one cannot separate the unconscious suppression phase that precedes target detection and the conscious reaction phase that follows target detection [42,58]. Faster responses to targets at certain locations of the visual field might therefore result from enhanced processing in either of these phases. Whereas the present results convincingly show that the upper nasal hemifield location speeds up the detection of visual targets during dichoptic stimulation, further investigation with other CFS techniques is needed to tease apart the effects of the pre and post perceptual processing phases and validate the prevalence of the upper nasal hemifield bias in other types of dichoptic stimulation tasks.

### Auditory facilitation of visual processing during CFS

Simple sounds combined with target occurrences reduced the time required for target detection during CFS, showing an advantage of approximately 130 ms over non-sound targets. However, no support for a crossmodal pitch-elevation compatibility effect was found, as high and low pitch tones similarly affected the RTs in the upper and lower visual field. This finding suggests that pitch-elevation mapping, which facilitates visual processing under standard viewing conditions [34,36–38], cannot be utilized in the processing of suppressed targets under dichoptic viewing.

In contrast to the present finding, some previous b-CFS studies have indicated that there are faster responses to suppressed visual targets compatible with simultaneously presented auditory stimuli [43,44]. Alsius and Munhall [43] found that a suppressed talking face video broke through suppression faster when combined with a voice speaking a sentence that matched (rather than mismatched) the lip movements of the talking face. Furthermore, Yang and Yeh [44] showed that discrete sound signals increased the speed of detection of suppressed visual targets when the sound was spatially congruent with the target (i.e., presented at the same depth plane via loudspeakers), but not when the sound was incongruent (i.e., presented at different depth plane via headphones). The most likely explanation for the discrepancy between the results of these studies, indicating crossmodal integration under CFS, and ours, showing no crossmodal congruency effect, is the type of audiovisual stimuli employed in the experiments. A talking face is a naturalistic stimulus that people are continuously exposed to and whose perception and understanding strongly depends on the quality of the audio-visual integration. This integration might therefore operate even between a suprathreshold auditory stimulus and preconsciously processed visual signal during CFS [43]. The spatial compatibility of a suppressed visual target to a suprathreshold auditory tone might similarly enable audio-visual integration at an early preconscious level [44]. By contrast, the pitch-elevation compatibility effect and other crossmodal effects based on the associative-semantic integration of abstract audio-visual features (see [34]) might require conscious processing of stimuli and hence not be easily observed in CFS tasks.

One could ask whether a larger spatial separation between targets in the upper and lower halves of the visual field might have amplified the pitch-elevation congruency effect. The distance between upper and lower targets (6.5. deg center-to-center distance) was adjusted here to ensure that a target breaking through suppression at either location could be detected while keeping the eyes at the center fixation cross; otherwise, the detection performance would have been confounded by eye movements. The study by Evans and Treisman [37] demonstrated that under standard viewing conditions, even a small spatial separation between upper and lower targets (6 deg) created a pitch-elevation congruency effect. It should be noted, however, that the results of the two studies are not necessarily fully comparable, as performance under standard viewing conditions is based on supraliminal perception, whereas both subliminal and supraliminal processes were likely involved in the present b-CFS task.

Similar to our findings, a study by Moors et al. [46] found no evidence of the influence of congruent auditory tones on the detection of visual looming targets that were suppressed from view with CFS. By measuring the contrast thresholds for target detection, the researchers showed that visual looming stimuli resulted in lower thresholds than a simple static grating, but auditory tone pips that were matched with the cycle of the looming stimulus did not further enhance performance. Based on their results, Moors et al. [46] concluded that certain forms of multisensory integration are not evident when the visual stimulus is suppressed from awareness. In the same vein, the lack of a pitch-elevation effect in the present study suggests that CFS, or dichoptic viewing in general, might limit the access of visual stimuli to the processing levels at which the binding of associative crossmodal information could occur. As a consequence, observers were able to benefit from sounds used as general detection cues for suppressed visual targets, but the subtler elements of crossmodal stimuli, such as pitch-elevation compatibility, could not be utilized.

It should be noted that the pitch-elevation congruency effect might have been diluted by non-optimal timing between the visual and auditory stimuli. Auditory tones were delivered 400 ms after visual target onset, at which point the target, gradually increasing in contrast, reached the 10% contrast level. This level was selected based on a pilot study, as most subjects were able to detect the target at this point. However, because of the simultaneity of stimulus presentation, a prerequisite for crossmodal integration to occur [32], could not be unequivocally determined by the combination of the subliminal visual targets and supraliminal discrete sounds, further studies should be carried out to clarify whether jittering the presentation time of the auditory stimulus or individually adjusting the stimulus timing would better support the crossmodal binding of associative audiovisual stimuli under dichoptic stimulation.

Nevertheless, as already stated, we found an RT advantage in sound-present trials independent of the target location and tone pitch. This finding is in line with previous studies that showed that sound stimuli can either enhance the processing of all visual stimuli [59] or act as warning signals for a change to occur in the visual field [60,61]. In other words, any co-occurring sound might have increased the saliency of a visual target within its dynamic background or improved the observer’s alertness by signaling the presence of a target before its detection, which might, in turn, also impair the observation of any potential audiovisual correspondence effects.

Interestingly, it appears that auditory stimuli played another important role, as the presence of sounds sped up learning during the course of the experiment. Observers who first ran b-CFS blocks containing auditory stimuli demonstrated shorter overall RTs than observers who first ran b-CFS blocks without auditory stimuli. The former group of observers showed faster target detection in the no-sound block presented after the sound-present blocks. These findings are consistent with previous research showing that multisensory stimulation facilitates visual learning [62], and such enhancements may emerge even within a single experimental session [63]. As such, the present results add to previous knowledge by showing that multisensory learning may also occur under dichoptic stimulation.

### Practical implications

Because dichoptic stimulation techniques are increasingly utilized as a means to manipulate visual awareness for various research purposes, understanding the visual asymmetry characteristics of dichoptic viewing are also of practical relevance. The upper nasal hemifield bias found in the present study calls for careful consideration of location-related effects and design features, particularly in CFS experiments, as the target location in such experiments is often employed as a response criterion [17,44,47,64,65]. Therefore, knowledge of the specific biases characteristic of dichoptic stimulation helps one to control unwanted locational effects and reduce their impact on the interpretation of results.

Furthermore, the findings are of practical value for novel display devices, such as monocular head-mounted displays (HMDs) that present display information to one eye while the other eye views the surrounding environment. Binocular rivalry is a common concern in these devices, and uncontrollable perceptual alternation between the two eyes’ views can compromise the user’s performance in any ongoing task [66–68]. Positioning the display in the user’s visual field so that the most critical information for a given task is available in the upper nasal visual field could alleviate rivalry alternation. Finally, based on our findings, crossmodal auditory cueing provides a potential means to influence visual target detection during dichoptic viewing.

## Conclusions

Suppressed visual targets during dichoptic viewing are most quickly detected when presented in the upper nasal hemifield of the eye. The target detection process can also be sped up by concurrent auditory tones, which seem to facilitate performance independent of the target location or associative tone pitch-target location congruency. These findings suggest that the processing of visual information under interocular suppression differs from that under standard viewing conditions in terms of location-based perceptual biases and crossmodal modulation of visual perception.

## Supporting information

S1 Data Dataset and description of the variables. (XLSX)

S1 Table Mixed-design ANOVA. (DOCX)
