# Supplementary material for: Upper nasal hemifield location and nonspatial auditory tones accelerate visual detection during dichoptic viewing
Source: PLoS One. 2018 Jul 23;13(7):e0199962. doi: 10.1371/journal.pone.0199962 (PMC6056051; doi:10.1371/journal.pone.0199962)
Supplement: S1 Table — (DOCX) [file pone.0199962.s002.docx]

**S1 Table. Mixed-design ANOVA.** ANOVA table with Location, Eye and Sound as within-subjects factors, and Dominance and Order as between-subjects factors.

|  | ***df*** | ***F*** | ***p*** | ***η_p_^2^*** |
| --- | --- | --- | --- | --- |
| **Location** | **3** | **10.90** | **.000** | **.295** |
| Location x Order | 3 | 1.73 | .169 | .062 |
| Location x Dominance | 3 | .58 | .629 | .022 |
| Location x Order x Dominance | 3 | 1.25 | .297 | .046 |
| *Error* | 78 |  |  |  |
| **Eye** | **1** | **3.18** | **.086** | **.109** |
| Eye x Order | 1 | 6.93 | .014 | .210 |
| Eye x Dominance | 1 | 2.05 | .164 | .073 |
| Eye x Order x Dominance | 1 | .14 | .716 | .005 |
| *Error* | 26 |  |  |  |
| **Sound** | **1.16** | **9.55** | **.003** | **.269** |
| Sound x Order | 1.16 | 67.38 | .000 | .722 |
| Sound x Dominance | 1.16 | .03 | .891 | .001 |
| Sound x Order x Dominance | 1.16 | 5.20 | .009 | .167 |
| *Error* | 30.1 |  |  |  |
| **Location x Eye** | **1.53** | **21.44** | **.000** | **.452** |
| Location x Eye x Order | 1.53 | 1.62 | .213 | .059 |
| Location x Eye x Dominance | 1.53 | .11 | .845 | .004 |
| Location x Eye x Order x Dominance | 1.53 | .44 | .596 | .017 |
| *Error* | 39.8 |  |  |  |
| **Location x Sound** | **4.05** | **.59** | **.671** | **.022** |
| Location x Sound x Order | 4.05 | .73 | .575 | .027 |
| Location x Sound x Dominance | 4.05 | .31 | .873 | .012 |
| Location x Sound x Order x Dominance | 4.05 | 1.09 | .364 | .040 |
| *Error* | 105 |  |  |  |
| **Eye x Sound** | **2** | **2.38** | **.102** | **.084** |
| Eye x Sound x Order | 2 | .16 | .854 | .006 |
| Eye x Sound x Dominance | 2 | .23 | .799 | .009 |
| Eye x Sound x Order x Dominance | 2 | .34 | .712 | .013 |
| *Error* | 52 |  |  |  |
| **Location x Eye x Sound** | **6** | **1.68** | **.130** | **.061** |
| Location x Eye x Sound x Order | 6 | 0.59 | .735 | .022 |
| Location x Eye x Sound x Dominance | 6 | 0.92 | .483 | .034 |
| Location x Eye x Sound x Order x Dominance | 6 | 1.35 | .238 | .049 |
| *Error* | 156 |  |  |  |
